# Human bone marrow mesenchymal stromal cells exhibit suppressive effects on lymphocytes derived from HTLV-1 infected individuals

**DOI:** 10.1186/1742-4690-12-S1-P9

**Published:** 2015-08-28

**Authors:** Evandra Strazza Rodrigues, Mayra Dorigan De Macedo, Katia Kaori Otaguiri, Maristela Delgado Orellana, Maurício Cristiano Rocha-Junior, Osvaldo Massaiti Takayanagui, Svetoslav Nanev Slavov, Dimas Tadeu Covas, Simone Kashima

**Affiliations:** 1 Regional Blood Center of Ribeirão Preto, Faculty of Medicine of Ribeirão Preto, University of São Paulo (USP), Brazil; 2 Faculty of Pharmaceutical Sciences of Ribeirão Preto, USP; 3 Department of Internal Medicine, Faculty of Medicine of Ribeirão Preto, USP, Brazil

## 

Human multipotent mesenchymal stromal cells (MSC) display immunoregulatory functions that can modulate innate and adaptive cellular immune responses. The suppressive and immunomodulatory activities of MSC occur by the action of soluble factors that are constitutively produced and released by these cells or alternatively, after induction of MSC stimuli in inflammatory microenvironments. During the infection by the human T cell lymphotropic virus-type 1 (HTLV-1) around 1-5% of the infected individuals will develop a chronic inflammatory disease known as HTLV-1-associated myelopathy/tropical spastic paraparesis (HAM/TSP). However, up to date it is unknown the contribution of the MSC in the inflammatory microenvironment resulting from this chronic disease. In this study, we evaluated the MSC immunosuppressive effect on HTLV-1 infected T lymphocytes. Assays using co-culture of MSC and HTLV-1+ T lymphocyte lineages resulted in a decrease of both tax gene expression and HTLV-1 p19 antigen. The reduction of the tax gene expression and the HTLV-1 p19 were at the same time associated with 1.7 fold increase of IL-6 secretion and a higher PGE2, IDO and VCAM-1 gene expression (p≤0.05). To evaluate if MSC immunoregulation can influence the proliferation of HTLV-1 infected T lymphocytes, we compared the proliferation of lymphocytes obtained from HTLV-1+ and healthy individuals co-cultured in the presence of MSC. It was observed that the lymphoproliferative inhibition by MSC on infected lymphocytes was similar compared to the cells obtained from healthy individuals. Additionally, this suppressive effect was related to a significant increase of IDO and PGE2 gene expression (p≤0.05). Furthermore, the HTLV-1 pol gene and p19 protein were less expressed after co-culturing with MSC, suggesting that the MSC immunoregulation is effective on HTLV-1 infected T cells. In conclusion, this study suggests that MSC could be involved in the immunomodulation of the HTLV-1 infected T lymphocytes. Financial Support: FAPESP, CNPq and FUNDHERP. Keywords: MSC, HTLV-1, immunoregulation, gene expression.

